# Predicting Adolescents’ Leisure-Time Physical Activity Levels: A Three-Wave Prospective Test of the Integrated Model of Self-Determination Theory and the Theory of Planned Behavior

**DOI:** 10.3390/bs14080693

**Published:** 2024-08-09

**Authors:** Diana L. Y. Su, Alison W. L. Wan, Lei Zhang, Jun Teng, Derwin K. C. Chan

**Affiliations:** 1 Department of Early Childhood Education, The Education University of Hong Kong, Hong Kong, China; dianasu@eduhk.hk (D.L.Y.S.);; 2 PE Department, Renmin University of China, Beijing 100872, China; 3 Institute of International and Comparative Education, Beijing Normal University, Beijing 100875, China; tengjun1983@bnu.edu.cn

**Keywords:** autonomous motivation, decision-making factors, social cognition beliefs, school health, after-school sports activities

## Abstract

A three-wave prospective study was conducted to provide a better understanding of the ability of the integrated model of self-determination theory (SDT) and the theory of planned behavior (TPB) to predict future physical activity (PA) engagement among adolescents. Nearly 2500 secondary school students from China were recruited to test the hypothesized pathway from autonomous motivation from SDT at baseline (T1) through the constructs of TPB one month later (T2) on leisure-time PA levels of secondary school students three months later (T3). The findings revealed that the structural equation models yielded excellent fit indices with χ^2^ = 1858.989, *df* = 257, CFI = 0.936, TLI = 0.926, RMSEA = 0.050 [90% CI = 0.048 to 0.052], and SRMR = 0.032. In particular, autonomous motivation at T1 was positively associated with attitude (R^2^ = 0.160), subjective norms (R^2^ = 0.160), and perceived behavioral control (PBC) (R^2^ = 0.173) at T2 (*β* = 0.395 to 0.414, *p* < 0.001) and subsequently associated with intention at T2 (R^2^ = 0.875, *β* = 0.112 to 0.478, *p* < 0.001). T2 intention was positively associated with leisure-time PA levels (R^2^ = 0.004) at T3. Our findings contribute to a better understanding of the motivational mechanisms and social cognition processes involved in predicting adolescents’ leisure-time PA levels among adolescents.

## 1. Introduction

Regular physical activity (PA) promotes both physical and mental well-being. However, it is concerning that over 80% of adolescents do not achieve the recommended levels of physical activity globally [1]. Driven by recognition of the importance of physical activity for health and well-being [2,3,4], as well as the alarming prevalence of insufficient PA levels among adolescents [5,6,7], researchers have employed psychological theories and models to explain the underlying psychological and behavioral processes that drive individuals’ engagement in PA [8,9,10,11]. The self-determination theory (SDT) [12,13] and the theory of planned behavior (TPB) [14] are two different psychological frameworks that are commonly used in the prediction [11,15,16] and promotion [17,18] of PA behaviors. An increasing number of studies have integrated these theories to provide a comprehensive understanding and explanation of individuals’ motivational and social cognition processes in the engagement of health-related behaviors [11,19]. This three-wave prospective study examined the sequence of the integrated model of SDT and TPB, revealing the extent to which autonomous motivation from SDT and social cognition constructs from TPB are predictive of secondary school students’ future leisure-time PA levels.

### 1.1. The Integrated Model of SDT and TPB

The theoretical integration of SDT [12,13] and TPB [14] recognizes both the humanistic motivational drive and the social cognitive process underlying human behaviors. The integrated theory, therefore, aims to combine the merits of both theories and resolve the theoretical boundaries of applying SDT or TPB alone in explaining human behavior [11,19]. Researchers have suggested that social cognition constructs from TPB could mediate the relationship between motivation orientation and particular behaviors [8,20]. Therefore, in the integrated model, it is speculated that autonomous motivation from the SDT functions as an antecedent to the social cognition constructs (i.e., attitudes, subjective norms, and perceived behavioral control (PBC)) from TPB, which in turn are associated with an individual’s intention to perform a particular behavior and future engagement [11,21,22].

The theoretical framework of the integrated model aligns with SDT prediction [13] that individuals’ motivational orientations stem from the satisfaction of inner psychological needs, leading to more favorable behavioral patterns. Integrating TPB can enhance the interpretative capability of SDT by clarifying how autonomous motivation delineates proximal social decision-making processes and manifests in behavior [20]. This integration may provide deeper insights into how individuals develop intentions that align with their underlying motivations, which are explained by the decision-making processes, subsequently guiding their intentions on future favorable behaviors.

The application of the integrated model has been developed and supported across multiple healthy behaviors, particularly in sports and PA participation [23,24,25]. However, previous studies have primarily utilized a cross-sectional, one-month prospective or longitudinal design to evaluate the relationship between the constructs of the integrated model in PA-related settings [19,26]. This approach might not be able to sufficiently test the stability and predictive ability of the integrated model among the SDT and TPB constructs and future behavioral patterns. Therefore, prior research might not be useful in providing explanations on how the constructs in the integrated model can predict future leisure-time PA levels among adolescents [27]. Moreover, a few studies have utilized the actual PA level as the outcome variable to examine the model because most studies focus solely on measuring the intention construct [9,25]. This may pose the limitation of being unable to explain why there is a discrepancy between intention and behavior using the TPB variables. Therefore, it is important to fill these research gaps in the existing literature.

### 1.2. The Present Study

The objective of this study was to employ the integrated model of SDT and TPB to test the theoretical sequence from autonomous motivation from SDT through constructs from TPB to predict the future leisure-time PA levels of secondary school students using a three-wave prospective design in Beijing, China. Drawing on the empirical evidence supporting the integration of SDT and TPB [23,24,25,28], it is expected that the results would provide insights into the motivational mechanisms and cognitive processes involved in adolescents’ engagement in PA and offer empirical support for the integrated theoretical framework.

Thus, we hypothesized the following pathways of the integrated model:

**H1:**  *Students’ autonomous motivation at T1 would be positively and significantly associated with the three social cognition beliefs (i.e., attitude, subjective norms, and PBC) at T2.*

**H2:**  *The three social cognition beliefs at T2 would be positively and significantly associated with intention toward PA engagement at T2.*

**H3:**  *Intention toward PA engagement at T2 would be positively and significantly associated with leisure-time PA levels at T3.*

We hypothesized that these predictions would hold after controlling for students’ age and sex.

## 2. Materials and Methods

### 2.1. Participants and Procedures

We recruited 2482 students (*M* age = 13.96, *SD* = 0.817; age range = 11 to 18 years; female = 50%) from 7 secondary schools across different regions of Beijing, China. The research protocol was approved by the Institutional Review Board of Education University of Hong Kong (Ref. No.: 2019-2020-0153). The inclusion criteria for the sample were (1) full-time secondary school students, (2) adolescents under the age of 18, (3) able to read and write Chinese to complete the questionnaire, and (4) no known disability, disease, or health condition that would affect PA participation. Informed consent forms were signed by the participants and their parents or legal guardians, ensuring their understanding of the children’s rights to participate in the study. An online questionnaire was designed to assess secondary school students’ autonomous motivation, social cognition beliefs, intention toward PA engagement, and leisure-time PA levels and was distributed at three time points: baseline (T1), one-month follow-up (T2), and three-month follow-up (T3). Responses were linked with unique identifiers to protect the participants’ confidentiality and anonymity. The study achieved a high retention rate, with 99.8% of participants completing the follow-up at T2 and 99.7% at T3.

### 2.2. Measures

Autonomous motivation: Autonomous motivation was measured by six items from the subscale of the simplified Chinese version [22] of the Treatment Self-Regulation Questionnaire (TSRQ) [29]. This scale has demonstrated robust psychometric properties and predictive power in PA settings [30,31]. An example item was “I want to do leisure-time physical activity because I have carefully thought about it and believe it is very important for many aspects of my life”. Participants responded to each item on a 7-point Likert scale with anchors ranging from “not at all true” (1) to “very true” (7). The Cronbach’s alpha of the construct at T1 was satisfactory (α = 0.876).

TPB variables: We used the physical activity version [32] of standard TPB measures [33] to assess attitude, subjective norms, PBC, and intention toward engaging in leisure-time PA. An example item was “I plan to do physical activity in my leisure time in the forthcoming month”. Participants responded on a 7-point scale with anchors ranging from “not at all true” (1) to “very true” (7). The Cronbach’s alphas of the TPB constructs at T2 were satisfactory (α = 0.876–0.962).

Leisure-time PA levels: We measured the levels of leisure-time PA using the short form of the International Physical Activity Questionnaire (IPAQ-SF) [34] at T3. Previous research has shown that, compared to objectively assessed PA, the IPAQ-SF exhibits adequate reliability and validity [35,36]. The IPAQ has been widely used and validated across diverse cultures, including Chinese populations [37,38], and has been shown to be valid among children and adolescents [39,40,41]. Participants were asked to report the frequency and duration of their PA in terms of “how many days in a week” and “how much time per day” they spent on vigorous and moderate-intensity PA, as well as walking, over the past seven days. We then calculated the scores of the IPAQ into the metabolic equivalent of task (MET) minutes/week following the guidelines of the IPAQ research committee [42].

### 2.3. Analysis

Zero-order correlations between the model constructs and Cronbach’s alpha reliability coefficient for the scales were analyzed using IMB SPSS Statistics 26. We used Mplus version 8.1 with robust maximum likelihood estimation to conduct confirmatory factor analysis to examine the factorial validity of the key psychological measures in our study (i.e., autonomous motivation, social cognition beliefs, and intention) and structural equation modeling to examine the sequence of proposed associations between autonomous motivation at T1, TPB variables at T2, and future leisure-time PA levels at T3 [43]. The raw MET scores, which were used as an outcome variable, were standardized to Z scores. Student’s age and sex served as confounding factors and were included as additional predictors in the model. Multiple goodness-of-fit indices (i.e., the comparative fit index (CFI), the Tucker–Lewis index (TLI), the root mean square error of approximation (RMSEA), and the standardized root mean square residual (SRMR)) were used to assess the overall fit of the proposed models. Models were considered to have acceptable goodness of fit if CFI and TLI values were close to or greater than 0.90, with RMSEA and SRMR values less than 0.08 [44].

## 3. Results

**Figure 1:**
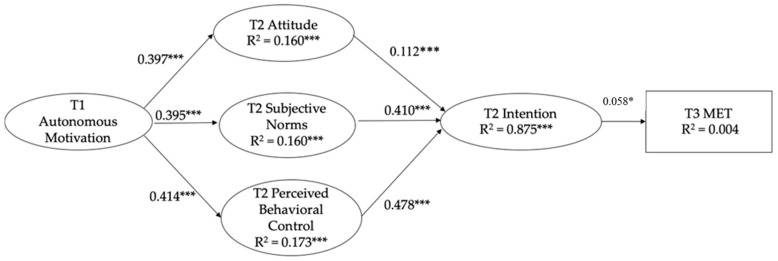
Standardized parameter estimates for SEM analysis. Parameter estimates of the SEM controlling for participant’s age and sex. * *p* < 0.05, *** *p* < 0.001.

The factorial validity of the measurement model, as shown in our confirmatory factor analysis, was excellent (χ^2^ = 1563.691, df = 199, CFI = 0.937, TLI = 0.926, RMSEA = 0.053 [90% CI = 0.050 to 0.055], SRMR = 0.031). The proposed models yielded excellent fit to the data (χ^2^ = 1858.989, *df* = 257, CFI = 0.936, TLI = 0.926, RMSEA = 0.050 [90% CI = 0.048 to 0.052], SRMR = 0.032). In support of H1, autonomous motivation at T1 was positively and significantly associated with attitude (R^2^ = 0.160), subjective norms (R^2^ = 0.160), and PBC (R^2^ = 0.173) at T2 (*β* = 0.395–0.414, *p* < 0.001). In support of H2, the cognition beliefs at T2 were positively and significantly associated with intention at T2 (R^2^ = 0.875) (*β* = 0.112 to 0.478, *p* < 0.001). In support of H3, there was a positive and significant relationship between intention at T2 and leisure-time PA levels at T3 (R^2^ = 0.004) (*β* = 0.580, *p* < 0.05) (see Figure 1 for the full standardized parameter estimates). The zero-order correlation matrix of the study variables is shown in Table 1.

## 4. Discussion

The present study aimed to adopt a multi-theory approach (i.e., the integrated model of SDT and TPB) to examine the sequence of the proposed associations between autonomous motivation from SDT, three cognition beliefs (i.e., attitude, subjective norms, and PBC) and intention from TPB, and future leisure-time PA levels among adolescents. By employing a three-wave prospective study design, our research could provide deeper insight into the predictive power of the integrated model for adolescents’ long-term leisure-time PA levels. This offers a more comprehensive understanding than earlier cross-sectional studies [24,25,45] that investigated the psychological determinants of PA behaviors. Overall, the findings of this study support the robustness of the integrated model in explaining how autonomous motivation and TPB constructs may predict future PA levels.

In line with our hypothesis (H1), the secondary school students’ autonomous motivation from SDT at T1 was significantly and positively associated with the three cognition beliefs (i.e., attitudes, subjective norms, and PBC) from TPB at T2. Consistent with previous findings, students with greater autonomous motivation to engage in PA were more likely to have positive attitudes toward PA, receive support from their significant others, and believe that they have the capacity to perform PA [46,47,48]. As such, when promoting PA engagement in young students, intervention strategies may aim to foster autonomous motivation by encouraging the internalization of behaviors, which in turn enhances positive social cognition beliefs toward PA.

Our findings also supported H2 that the three cognition beliefs (i.e., attitude, subjective norms, and PBC) exhibited significant path prediction of intention toward PA engagement. These results are in line with the proposed tenet of TPB [14]. Furthermore, these findings are also consistent with previous research [10,47,48,49], which revealed that students with greater intentions toward PA engagement endorsed more favorable attitudes toward PA, received support to engage in PA from their significant others, and perceived the ease of performing PA. However, the findings of the study of Hagger et al. (2009) [47] did not support the path from subjective norms on intention, as subjective norms were not related to intentions toward engagement in leisure-time PA among adolescents in Estonia and Finland. One possible explanation for the positive relationship between intention and subjective norms might be that students’ acceptance of and deference to the expectations of significant social agents might be seen as encouraging and consistent with their own principles [50]. Indeed, our speculation regarding the probable reason for cultural differences would require further studies with samples from multiple cultural groups.

Consistent with H3, intention toward PA engagement at T2 was positively related to leisure-time PA levels at T3. This indicates that a stronger intention toward PA engagement among adolescents could be predictive of their future leisure-time PA levels. This finding is in line with a meta-analysis [51] and other studies [9,22,48] that demonstrated a positive association between intention and students’ PA levels. This finding is also consistent with the proposed path of TPB, in which intention is considered the most proximal determinant of individuals’ behavioral engagement. However, the effect observed in our study was relatively small (R^2^ = 0.004). This suggests that intentions toward PA engagement might not provide a strong prediction of the future leisure-time PA levels among adolescents, limiting the ability of intentions to predict future behavioral patterns. Apart from the fact that the PA level in our study was a follow-up assessment of 3 months, which was relatively longer than that of other previous studies [19,26], the small variance of PA level in our study may be explained by the widely recognized influence of academic stress, academic burden, and examination-related stress on adolescents in China, which might significantly undermine their participation in actual leisure-time activities, including exercise and sports [52].

### 4.1. Limitations and Future Directions

Although our study has the strengths of having a large sample size and utilizing a three-wave prospective design, it is important to acknowledge certain methodological and theoretical limitations that can provide insights for future research.

First, the leisure-time PA level in our study was measured by a self-reported survey, so the responses of participants could be subject to response bias [53]. Although research has revealed that there is a moderate to high correlation between self-reported and objective measures of PA levels [54], future studies may consider utilizing objective measures, such as accelerometers, to assess the PA levels of adolescents. Second, previous studies have identified potential moderators of the integrated model, such as personal PA habits, personality traits (i.e., self-control and extraversion/neuroticism), environmental factors (i.e., social influence and environmental proximity), and cultural influences [32]. Future studies could investigate the moderating effects of these variables on the pathways of the integrated model to enhance our understanding of individuals’ PA behavioral patterns. Third, although the three-wave prospective design of this study enabled testing of the temporal sequence within the proposed integrated model to predict future behavioral patterns, our findings did not allow us to draw absolute conclusions about the causal and temporal relationships between the integrated model constructs and future leisure-time PA levels. Future studies should employ a longitudinal study design, such as a three-wave cross-lagged panel design, to rigorously test these causal relationships [22]. Fourth, as our student sample came from seven secondary schools in Beijing, the hierarchical nature of the nested data could have led to underestimated standard errors, inflated Type I error rates, and potentially biased parameter estimates [55]. Future studies could adopt multilevel modeling methods and the summary-statistics approach to analyze nested data [56]. Finally, our sample was limited to children from a single country, preventing us from making cross-cultural comparisons among children from various cultures or geographical regions. Future research should include samples from both Western and Eastern cultures to enhance the generalizability of the findings related to the integrated model in sports [48].

### 4.2. Conclusions and Implication

The current study utilized a three-wave prospective design to examine the extent to which the constructs within the integrated model of SDT and TPB can predict future leisure-time PA levels among adolescents in China. Our findings can offer a better understanding of the motivational mechanisms and social cognition processes influencing leisure-time PA levels among adolescents. In line with the hypothesized model, the findings showed that autonomous motivation at T1 was positively associated with three social cognition beliefs and intention toward PA engagement at T2, and intention was subsequently positively associated with leisure-time PA levels at T3. Therefore, the findings suggest that adolescents with greater autonomous motivation are more likely to develop positive attitudes, receive social support for PA engagement, believe in their capability to perform PA, and have stronger intentions toward PA engagement, thereby predicting higher levels of leisure-time PA among adolescents.

## Figures and Tables

**Table 1:** Zero-order correlations and Cronbach’s alphas of study variables.

Variables	1	2	3	4	5	6
1. T1 Autonomous motivation	*-*					
2. T2 Attitude	−0.158 **	*-*				
3. T2 Subjective norms	−0.109 **	0.536 **	*-*			
4. T2 PBC	−0.165 **	0.635 **	0.756 **	-		
5. T2 Intention	−0.185 **	0.654 **	0.795 **	0.849 **	*-*	
6. T3 Leisure-time PA levels	−0.017	0.043	0.068 **	0.084 **	0.054 *	-
Mean	5.677	6.140	5.534	5.663	5.739	2494.219
S.D.	1.069	1.124	1.283	1.231	1.255	2586.483
Cronbach’s alphas	0.876	0.962	0.876	0.953	0.943	-

* *p* < 0.05 ** *p* < 0.01.

## Data Availability

Data is available upon request due to ethical considerations and privacy consent.
